# Health-related quality of life of people living with HIV in Beni-Mellal City, Morocco

**DOI:** 10.1093/inthealth/ihaf022

**Published:** 2025-03-21

**Authors:** Mina Benaddi, Abdelhafid Benksim, Touria Fatihi, Sanaa Sabour Alaoui

**Affiliations:** Polyvalent Research and Development Team Polydisciplinary Faculty, The University of Sultan Moulay Sliman Beni Mellal, Biological Department, Beni Mellal 23000, Morocco; Regional Health Directorate, Higher Institute of Nursing Professions and Health Techniques (ISPITS-M), Nursing Care Department, Marrakech 40000, Morocco; Department of Infectious Diseases, Regional Hospital of Beni Mellal, Beni Mellal 23000, Morocco; Polyvalent Research and Development Team Polydisciplinary Faculty, The University of Sultan Moulay Sliman Beni Mellal, Biological Department, Beni Mellal 23000, Morocco

**Keywords:** HIV/AIDS, HRQoL, PLHIV

## Abstract

**Background:**

Nowadays, the objective for people living with HIV (PLHIV) is living well and long with the infection rather than surviving it. This study assesses health-related quality of life (HRQoL) of PLHIV in Beni-Mellal City, Morocco, and the factors influencing HRQoL.

**Methods:**

A cross-sectional study was conducted on 160 PLHIV attending the regional hospital of Beni-Mellal, Morocco. HRQoL was assessed using the EQ-5D Questionnaire (EuroQol-5D). A non-probabilistic χ^2^ test was used to determine factors associated with HRQoL of PLHIV. A multivariate binomial logistic regression model was applied to determine potential factors influencing HRQoL.

**Results:**

The rate of PLHIV with good HRQoL (≥0.59 EuroQol-5D score) was 64.4%. The factors associated with good HRQoL were male gender (p=0.026) (OR: 3.69; 95% CI 1.0 to 12.135), side effects (p= 0.006) (OR: 3.655; 95% CI 1.446 to 9.239), high CD4 count (p=0.001) (OR: 0.77; 95% CI 0.061 to 0.190), HIV representation (p=0.01) (OR: 7.923; 95% CI 1.65 to 38.04), good relationship with the healthcare team (p=0.002) (OR 5.771; 95% CI 1.0 to 12.135) and low viral load (p=0.001) (OR: 0.180; 95% CI 0.063 to 0.514).

**Conclusions:**

Psychological support and challenging stigma are key in health strategies to improve the HRQoL of PLHIV.

## Introduction

In 2020, 1.5 million new HIV infections and 680 000 deaths related to HIV were registered globally.^1^ Nevertheless, the epidemic status of HIV has changed since the generalization of antiretroviral therapy (ART) in 1994. Currently, HIV infection is increasingly considered a chronic disease, as people living with HIV (PLHIV) who adhere to ART and achieve viral suppression have almost the same life expectancy as anyone else. Indeed, the 90–90–90 targets set by The Joint United Nations Program on HIV/AIDS (UNAIDS) have drastically improved the clinical outcomes of HIV infection.^2^ Consequently, the experience of living with HIV has gone from one of survival to one of living well and living long. Thus, the health-related quality of life (HRQoL) has been pointed out as the fourth 90 target to work on.^3^ The WHO has defined HRQoL as ‘individuals perceptions of their position in life in the context of the culture and value systems in which they live and in relation to their goals, standards, expectations and concerns’.^4^ Furthermore, the WHO strategy's central goal, fully articulated, is ‘to end the AIDS epidemic as a public health threat by 2030, within the context of ensuring healthy lives and promoting well-being for all at all ages’.^5^ Hence, viral load suppression is not a final goal to achieve among PLHIV, but rather a starting point to manage and cope with other aspects of physical, social and psychological difficulties facing PLHIV. The HRQoL could be associated with a set of determinants. According to the PRCEDE-PROCEED model, three categories of determinants interfere with lifestyle and health. The first category consists of the predisposing determinants (knowledge, attitudes), the second the enabling factors (healthcare affordability) and the third the reinforcing factors (social support).^6^ International reviews, of the quality of life among PLHIV, reveal several factors associated with HRQoL, such as income,^7–10^ gender,^11,12^ educational level,^7,10,13^ age,^11,13^ accessibility and satisfaction with healthcare services,^9^ length of living with the infection,^8^ comorbidities,^8,10^ social support^8,9^ and the existence of symptoms.^8,10,14^

In Morocco, the prevalence of HIV is well controlled among the general population, with a rate of 0.07%. Nonetheless, the prevalence among the key populations (sex workers, men having sex with men and users of injectable drugs) is more important. In 2022, >21 600 people were living with HIV, with 1359 new infections and 360 deaths related to AIDS-related illnesses.^15^ In the region of Beni-Mellal-Khenifra, the rate of HIV positivity in 2019 was 0.07.^16^ As there are a lack of data on the HRQoL in Morocco, this study aimed to identify the HRQoL level and its associated factors among PLHIV in the regional hospital of Beni-Mellal, Morocco. To the best of our knowledge, there are no other published reports on HRQoL in the Beni-Mellal-Khenifra region, so the results of this study would be relevant for the conception of healthcare strategies.

## Materials and methods

### Study design and sitting

This study was a cross-sectional study conducted from March to July 2022 at the infectious diseases department in the regional hospital of Beni-Mellal City, a city in the center of Morocco. The cited center serves >2.5 million people living in the five towns of the region of Beni-Mellal-Khenifra.

### Study participants

All the adult PLHIV, aged >18 y, attending the infectious diseases department in the regional hospital of Beni Mellal and being under an ART regimen for >2 mo, with no hearing problems or mental disorders, were included.

### Sampling procedure and sample size

The sample size was calculated using the representative sample formula: n=z² x p (1–p)/m² (Z=1.96 confidence level at 95%, p=proportion of population having the character, m=error marge tolerated). The assumption of a 95% level of confidence and a 5% margin of error was adopted. Consequently, a representative sample size of 160 (53.3%) people among 300 PLHIV under an ART regimen was collected. Participants were recruited using a non-probabilistic accidental sampling, adaptable with specific groups with limited accessibility of all participants, and time and cost economizing. Thus, the data collected included every person living with HIV under an ART regimen attending the infectious diseases department for the control consultation during the survey until the achievement of the 160 PLHIV quota.

### Data collection

A structured self-designed interviewer-administrated questionnaire was used to collect data along with consultation of medical records. The independent variables were set up according to the PRECEDE-PROCEED model. The latest is a framework developed to assess the impact of healthcare actions on the quality of life. According to this model, the quality of life is studied in conjunction with predisposing, enabling and reinforcing factors. The first category was divided, in the current survey, into genetic, cognitive and sociodemographic factors such as sex, age and educational level (illiterate, primary, secondary, higher). The second consisted of essential healthcare services accessibility (<20, 20–60 and >60 km away from the facility), health insurance (with or without insurance), the quality of relationship with healthcare givers (satisfied or not), treatment regimen—first line (2 nucleoside reverse transcriptase inhibitor [NRTI] + 1 non-nucleoside reverse transcriptase inhibitor [NNRTI]) or second line (2 NRTI + 1 NNRTI + 1 protease inhibitor)—viral load (detectable or non-detectable) and side effects (existing or not). Regarding the reinforcing factors, these constituted marital status (with or without spouse), occupation (formal [public servant or employee] or non-formal work [daily, periodic or no definite work]), sharing of HIV status (yes or no) and acceptance of the infection. The HRQoL (the dependent variable) was assessed using the Euroqol-5D-5 L measuring scale, whether good or low. The Europol-5D is a concise, generic measure of self-reported health accompanied by weights reflecting the relative importance to people of different health problems. The concept of health being measured by EQ-5D is variously described as health status or HRQoL. As the EQ-5D-5 L is a ‘generic’ tool, it can measure health across different sorts of patients, disease areas and treatments. Different researchers widely utilize this tool because it minimizes the burden of data collection, and can be used in various healthcare sector applications.^17^ The scale uses five dimensions for describing health states (5D): Mobility; Usual Activities; Self-care; Pain & Discomfort; and Anxiety & Depression. Each scale dimension has five levels explicitly expressed as no, mild, moderate, severe and extreme or unable to. Scale scores range from −0.59 to one.^17^ One is the best possible health state. Negative values represent health states perceived as worse than being dead (which is equal to 0). Alongside the EQ-5D, the EQ visual analog scale (VAS) (a 20 cm VAS) is used to give patients a possibility to assess their health with endpoints labeled ‘Best imaginable health state’ (100) and ‘Worst imaginable health state’ (0).^18^ The scores of the EQ-5D-5 L were calculated using a matrix developed by a Belgian team and categorized into two modalities: a good level (≥0.59) of HRQoL and a low level of HRQoL(≤0.58).^19^

### Data processing and analysis

Each item of the questionnaire was checked and coded before data entry. Without any missing registration, data were exported to IBM's Statistical Package for the Social Sciences (SPSS) version 21 software for data analysis. SPSS is software used for statistical analysis, data management and data documentation developed by IBM Corp., located in Armonk (N.Y., USA). Descriptive statistics of data were essentially about mean, mode and standard deviance (SD) for numerical variables and headcounts and percentages for categorical variables. To study the distribution of data, a Kolmogorov–Smirnov test was performed, which revealed that data were normally distributed with 0.296 mean and 0.38 SD. The univariate analysis used a one-sided χ^2^ test to recognize associations between the independent and dependent variables. Therefore, all the factors identified in the univariate analysis with p<0.20 were used within a multivariate binary logistic regression model to determine potential factors influencing the HRQoL among PLHIV. The OR coefficient and its 95% CI were used to estimate the associations. Adjustment of confounders was addressed by using a multivariate analysis in the regression model.

## Results

### HRQoL by predisposing, enabling and reinforcing factors

All the 160 participants in the study fully responded to our questionnaire (100% response rate). The descriptive statistics (mean, mode, SD and variance for numeric variables; headcount and percentage for categorical variables) revealed that the mode age of participants was 36 y, the dominant gender was female (67.5%), the majority of participants were from rural areas (70.6%) and married (56.9%). For the economic characteristics, 41.2% of the participants declared having no stable work, 58.4% having no health insurance and 40% were illiterate (Table 1).

**Table 1.** Health-related quality of life (HRQoL) among people living with HIV by predisposing, enabling and reinforcing factors

Sociodemographic characteristic	HRQoL		p
	Good 103 (64.4%)	Low 57 (35.6%)	
Age range (y)			
18–45	82 (79.7%)	39 (68.4%)	0.256
45–65	19 (18.4%)	17 (29.8%)	
>65	2 (1.9%)	1 (1.8%)	
Sex			
Male	26 (25.2%)	26 (45.6%)	0.08
Female	77 (74.8%)	31 (54.4%)	
Origins			
Urban	33 (32.0%)	14 (24.6%)	0.320
Rural	70 (68.0%)	43 (75.4%)	
Marital status			
With spouse	58 (56.3%)	33 (57.9%)	0.44
Without spouse	45 (43.7%)	24 (42.1%)	
Occupation			
Formal work	20 (19.4%)	11 (19.3%)	0.98
Informal work	83 (80.6%)	46 (80.7%)	
Medical insurance			
With health insurance	69 (67.0%)	45 (78.9%)	0.110
Without health insurance	34 (33.0%)	12 (21.1%)	
Educational level			
Low	58 (56.4%)	23 (40.4%)	0.23
High	9 (8.7%)	6 (10.6%)	
Illiterate	36 (34.9%)	28 (49.1%)	
Healthcare services accessibility			
<20 km	34 (33.0%)	17 (29.8%)	0.76
20–60 km	42 (40.8%)	22 (38.6%)	
>60 km	27 (26.2%)	18 (31.6%)	
Relationship with healthcare givers			
Satisfied	95 (92.2%)	39 (68.4%)	0.001
Not satisfied	8 (7.8%)	18 (31.6%)	
Type of treatment molecule			
First line	90 (87.4%)	48 (84.2%)	0.57
Second line	13 (12.6%)	9 (15.8%)	
Length of life with infection			
<5	38 (36.9%)	28 (49.1%)	0.26
5–10	55 (53.4%)	23 (40.4%)	
≥10	10 (9.7%)	6 (10.5%)	
Benefit of therapeutic education			
Yes	83 (80.6%)	38 (66.7%)	0.039
No	20 (19.4%)	19 (33.3%)	
Existence of side effects			
Yes	76 (73.8%)	34 (59.6%)	0.48
No	27 (26.2%)	23 (40.4%)	
Viral load			
Detectable	8 (9.8%)	19 (33.3%)	0.001
Undetectable	95 (92.2%)	38 (66.7%)	
Count of CD4			
<500	79 (76.7%)	16 (28.1%)	0.001
≥500	24 (23.3%)	41 (71.9%)	
Representation of HIV			
Chronic disease	11 (10.7%)	13 (22.8%)	
Fatal disease	43 (41.7%)	14 (24.6%)	0.003
Divine punishment	49 (4.6%)	30 (52.6%)	
Sharing of HIV status			
Yes/No	64 (62.1%)/39 (37.9)	43 (5.4%)/14 (24.6%)	0.06
Feeling social support			
No/Yes	16 (15.5%)/86 (84.4%)	12 (21%)/45 (79%)	0.004

Almost one-half of the population studied (48.8%) had been living with HIV for >5 y, 57.5% of them under a first-line ART regimen and 69% of them experiencing treatment side effects. For healthcare services accessibility, 72% of participants lived within a 60 km radius of the facility, 75.62% received patient therapeutic education and 82.7% were satisfied with their relationship with healthcare givers. Regarding the reinforcing factors, 49.4% of participants did not accept the infection and consider it a divine punishment, 66.9% of them had shared their HIV status with one of their relatives and 18% of them did not feel they had any social support (Table 1).

The univariate data analysis revealed that a good relationship with the healthcare team (p=0.001), benefit from therapeutic patient education (p=0.039), undetectable viral load (p=0.001), CD4 count (p=0.001), the feeling of social support (p=0.004), as well as acceptance and representation of HIV (p=0.003) were significantly associated with HRQoL (Table 1).

### Factors associated with ART adherence according to binary logistic regression

According to the binary logistic regression model, HRQoL among PLHIV was associated with sex (OR: 3.69; 95% CI 1.0 to 12.135), relationship with healthcare givers (OR: 5.771; 95% CI 1.0 to 12.135) and HIV representation (OR: 7.923; 95% CI 1.65 to 38.04). It also revealed a significant association with CD4 count (OR: 0.77; 95% CI 0.061 to 0.190), the existence of side effects (OR: 3.655; 95% CI 1.446 to 9.239) and viral load (OR: 0.180; 95% CI 0.063 to 0.514) (Table 2).

**Table 2.** The factors associated with health-related quality of life among people living with HIV according to the binominal logistic regression model

Associated factor	B	SE	Wald	p	Exp(B)	95% CI
Sex	1.327	0.597	4.945	**0.026**	3.769	[1.170–12.135]
Health insurance	0.938	0.586	2.563	0.109	2.554	[0.810–8.054]
The benefit of therapeutic patient education	−1.161	0.704	2.719	0.99	0.313	[0.79–1.245]
Viral load	17.16	4019.77	6.977	**0.001**	0.180	[0.063–0.514]
Side effects	1.296	0.473	7.506	**0.006**	3.655	[1.446–9.239]
CD4 count	−2.561	0.459	31.167	**0.001**	0.77	[0.031–0.190]
Relationship with healthcare givers	−1.753	0.579	9.180	**0.002**	5.771	[1.857–17.935]
HIV representation	2.070	0.800	6.692	**0.01**	7.923	[1.651–38.04]
Share of HIV status	0.652	0.458	2.030	0.154	1.920	[0.783–4.711]
The feeling of social support	−2.164	200034.954	11.296	0.096	0.362	[0.109–1.990]

The significance of the values has been stated in bold.

## Discussion

The current study aimed to describe the HRQoL and its related factors among PLHIV in the Beni-Mellal-Khenifra region. The participants in the current study were mostly females (67.5%), in contradiction with a survey conducted in western France where the dominant gender was male (75.2%).^8^ The current study's mean age was 40.71 y, which is higher than that in a study conducted in Brazil (37.6 y).^12^ The majority of the population studied came from rural areas (70%) and were married (56.9%), in concordance with a study in Indonesia where the majority of participants were married and from rural areas.^14^ In addition, most of the studied population were without stable work (80.2%), in discordance with a study conducted in Norway where only 30% of participants were unemployed.^13^ Regarding educational level, the majority of survey participants were illiterate (62%), with very low proportions of university-educated participants, unlike other studies conducted in the MENA (Middle East and North Africa) region).^7^ According to the questionnaire results, 83.8% of PLHIV were satisfied with the quality of the medical and nursing care, and 83.7% had an undetectable viral load.

Most participants (64.4%) reported a good level of HRQoL. This result is higher than the one found in a survey conducted on 965 participants in France (63%),^8^ whereas it was slightly lower than the rate found in a study conducted in Italy.^20^

Regarding the factors associated with HRQoL, the current study revealed that the male gender was more likely to report good HRQoL, which corroborates several studies.^11,12,21^ This influence of patient gender on HRQoL may be explained by the differences in men's and women's cultural and social conditions. Indeed, women in some cultural contexts had less access to healthcare services, information and education, while they are more subject to social stigma, violence and social duties. For the gender component, Morocco has notably participated actively in community dialogue with women living with or vulnerable to HIV, on the link between gender-based violence and HIV. However, further actions should be taken to enhance gender equity in the matter of sexual and reproductive health. Furthermore, women's social conditions (access to education, work and politics) should be improved to give them the necessary competencies to face challenging situations, as is the case for HIV infection.

Contrary to other reports, the current study revealed no significant association between age and HRQoL among PLHIV. This difference, between the results found in the current study and others, may be linked to the fact that the population in the present study is predominantly young (75.6% were aged 18–45 y), with a mode of 36 y, so there is no significant oscillation in age between participants that could affect the degree of compliance. Indeed, studies highlighting age as related to good HRQoL mostly involved participants aged >40 y. Thus, with age advancement and the emergence of dysfunctions, the perception of treatment benefits and disease risk and danger became more perceptible, which motivates ART adherence, acceptance of disease and social support, which may enhance the HRQoL.

Several studies have revealed that income and employment status were factors positively influencing HRQoL,^8,9,12,13^ whereas the current study showed no statistically significant difference in HRQoL between patients with formal work and stable income and those without any formal work. This discrepancy could be explained by the fact that the participants benefit from free healthcare and social assistance from thematic associations. Consequently, financial accessibility to healthcare services is not an issue that may lower the levels of HRQoL. On the other hand, the supportive family fabric in Moroccan society provides a source of funding and subsidies for needs, even for people with no stable income, which provides a feeling of security and safety from financial threats that could influence the HRQoL.

Another factor that emerged in other studies as having a significant influence on the HRQoL is the perception of side effects of treatment, as in a cross-sectional study carried out in Sichuan, China.^22^ The results of the current study corroborate these findings, as the participants experiencing undesirable side effects are less likely to report good HRQoL levels. The existence of treatment side effects exposes an individual to physical and emotional discomfort that affects their HRQoL. Consequently, governments should be encouraging the drug industry to develop more tolerated treatments, to guarantee a good level of HRQoL for PLHIV.

The current survey shows that undetectable viral load and CD4 count beyond 500 are positively associated with good levels of HRQoL. Indeed, the association between viral load and CD4 count and HRQoL is recurrent in the literature.^8,10,14^ The viral load suppression and the high counts of CD4 guarantee the absence of symptoms and opportunistic infections, which drastically increase the HRQoL in all its dimensions (physical, social, psychological and emotional). Good levels of CD4 counts and viral load suppression could not be achieved without an effective therapeutic adherence to antiretroviral therapy. Healthcare systems are asked to act on all factors (cognitive, financial, social and related to accessibility) enhancing therapeutic adherence to achieve viral suppression and maintain good levels of HRQoL among PLHIV.^23^

According to the OR values, those PLHIV who are satisfied with healthcare givers and healthcare services are 5.77 times more likely to have good levels of HRQoL. Thus, being pleased with healthcare services is a predictor of good HRQoL. Hence, health strategies should focus on the availability of and accessibility to quality healthcare services. Integration of systematic psychological support in healthcare services would be crucial to achieving high levels of HRQoL, due to the majority of participants presenting medium levels of depression and anxiety. Furthermore, integrating HIV healthcare at the primary healthcare level would be crucial for effective health service delivery, especially those aiming to reduce the low and heterogeneous screening coverage.

Multiple reports have pointed out that social support is positively influencing the HRQoL among PLHIV.^8,9,22^ In the current study, although the regression model did not show a significant association, the univariate analysis reported social support to be a factor associated with HRQoL. Indeed, PLHIV feeling socially supported had better HRQoL scores, especially in the dimension of anxiety and depression. Consequently, the reinforcement of the struggle against stigma is primordial.^24^ Indeed, the Moroccan national strategy on human rights in the context of HIV/AIDS insists on the necessity of erasing discrimination and ensuring psychosocial support for PLHIV.^25^ Nevertheless, the majority of planned actions target the care facilities, while broader actions of education and awareness of the different components of society should be engaged to work toward the zero-stigma goal. The struggle against stigma and discrimination is not a healthcare concern only; it is a multidimensional and multisectoral action that should be taken by all.

Finally, an individual’s beliefs and representation of HIV infection are reported to be significantly associated with HRQoL.^12^ Similarly, the current study shows that accepting HIV infection as a chronic disease and believing in the capacity to live well and long with it, affects one's quality of life, especially the aspect of depression and anxiety. Consequently, more actions should be taken to change negative beliefs. Two types of actions are needed: the first category is on behalf of the person infected, in terms of generalization and standardization of therapeutic patient education, as it leads to a better understanding of the infection and it gives the suitable psychological support needed to enhance infection acceptance; the second category concerns the community. Indeed, focused actions are needed to change the collective consciousness and negative attitudes by reinforcing educational programs about HIV, recurrent media spots about HIV and public conferences and discussion days to remove negative representations at their source, especially among young people.^26^

## Strengths and limitations

The cross-sectional study allows the examination of multiple factors at the same time without being time- and money-consuming. It also allows access to specific groups of participants, as is the case in the current study. Furthermore, the current study is one of the few, if non-existent, papers in this specific area of the national territory on this specific topic, which makes it an adequate basis for future research and policies. The sampling procedure and the sample size could be considered a limitation. Because the latter is not probabilistic, it increases the selection bias, such as less representativeness and under- or over-representing of certain viewpoints, which could explain certain wide CIs and limit the certainty of the results. Furthermore, there is a limit to the generalization of the findings, as the study type does not establish the cause-to-effect relation.

## Conclusion

The rate of PLHIV having good levels of HRQL, revealed by the current study, is below the 90% target of PLHIV with a suitable level of quality of life. The HRQoL is defined by several factors going beyond physical and biological aspects. Indeed, offering reliable care to PLHIV is about not only viral suppression and healing from opportunistic infections, it is also about guaranteeing them conditions for living well. Thus, government activities and healthcare strategies should give more importance to social and psychological aspects influencing the quality of life of PLHIV. Consequently, the involvement of all political, social and educational actors is required to achieve the fourth 90 target. Furthermore, healthcare givers have to be aware of those aspects, to offer holistic and comprehensive care to PLHIV. Further research is required to raise awareness about this specific and important dimension of the challenges facing PLHIV.

## Data Availability

The datasets used and analyzed during the current study are not publicly available, for the privacy and sensibility of the participants’ information.
